# Establishment and Characterization of Stable Zein/Glycosylated Lactoferrin Nanoparticles to Enhance the Storage Stability and *in vitro* Bioaccessibility of 7,8-Dihydroxyflavone

**DOI:** 10.3389/fnut.2021.806623

**Published:** 2022-01-03

**Authors:** Yufeng Chen, Xiaojing Gao, Shucheng Liu, Qiuxing Cai, Lijun Wu, Yi Sun, Guobin Xia, Yueqi Wang

**Affiliations:** ^1^College of Food Science and Technology, Zhejiang University of Technology, Hangzhou, China; ^2^Guangdong Provincial Key Laboratory of Aquatic Product Processing and Safety, College of Food Science and Technology, Guangdong Ocean University, Zhanjiang, China; ^3^College of Food Engineering, Beibu Gulf University, Qinzhou, China; ^4^Department of Pediatrics Section of Neonatology, Texas Children's Hospital, Houston, TX, United States; ^5^Key Lab of Aquatic Product Processing, Ministry of Agriculture and Rural Affairs of the People's Republic of China, South China Sea Fisheries Research Institute, Chinese Academy of Fishery Sciences, Guangzhou, China

**Keywords:** zein, glycosylated lactoferrin, nanoparticles, bioaccessibility, 7,8-dihydroxyflavone

## Abstract

In this work, the lactoferrin (LF) was glycosylated by dextran (molecular weight 10, 40, and 70 kDa, LF 10K, LF 40K, and LF 70K) *via* Maillard reaction as a stabilizer to establish zein/glycosylated LF nanoparticles and encapsulate 7,8-dihydroxyflavone (7,8-DHF). Three zein/glycosylated LF nanoparticles (79.27–87.24 nm) with low turbidity (<0.220) and polydispersity index (PDI) (<0.230) were successfully established by hydrophobic interactions and hydrogen bonding. Compared with zein/LF nanoparticles, zein/glycosylated LF nanoparticles further increased stability to ionic strength (0–500 mM NaCl) at low pH conditions. Zein/glycosylated LF nanoparticles had nanoscale spherical shape and glycosylated LF changed surface morphology of zein nanoparticles. Besides, encapsulated 7,8-DHF exhibited an amorphous state inside zein/glycosylated LF nanoparticles. Most importantly, zein/glycosylated LF nanoparticles had good water redispersibility, high encapsulation efficiency (above 98.50%), favorable storage stability, and bioaccessibility for 7,8-DHF, particularly LF 40K. Collectively, the above research provides a theoretical reference for the application of zein-based delivery systems.

## Introduction

An infrequent flavone monomeric compound existing in *Godmania aesculifolia* and *Tridax procumbens*, 7,8-dihydroxyflavone (7,8-DHF) (Supplementary Figure 1), was authenticated as a high-affinity tropomyosin receptor kinase B (TrkB) agonist (1, 2). It could mimic the physiologic roles of brain-derived neurotrophic factor (BDNF) and its downstream signaling pathway (3). Current literature confirm that 7,8-DHF can relieve a lot of BDNF-relevant human sickness, such as obesity, Parkinson's disease, Alzheimer's disease, and depression (4–8). However, methylation, sulfation, and glucuronidation of 7,8-DHF in the intestinal tract and liver caused its extremely low oral bioavailability (9). In our previous research, we testified that the permeability coefficient of 7,8-DHF was lower than 3 × 10^−6^ cm/s, and it had an active efflux mediated by multidrug resistance-related proteins (MRPs, especially MRP 2 outflow) and P-glycoprotein (P-gp) (10). Thus, the low chemical instability, low oral bioavailability, and high intestinal efflux of 7,8-DHF restricted its application as a functional component in the food industry.

Encapsulating 7,8-DHF within well-designed nano-sized carriers might be a promising alternative to overcome the chemical instability and low bioavailability in food industry. In our previous study, decorative liposomes were fabricated to encapsulate 7,8-DHF with improved stability, *in vitro* bioaccessibility, and permeability coefficient across Caco-2 cell monolayers (11). Recently, food-grade protein nanoparticles have been increasingly applied in encapsulating health-promoting naturally occurring flavonoids. For instance, epigallocatechin gallate and curcumin were encapsulated by zein and caseinate (12, 13). Particularly, zein has been the present research hotspot focus on hydrophobic flavonoids delivery by antisolvent precipitation (ASP) (14). Penalva *et al*. has fabricated zein nanoparticles for encapsulating resveratrol to improve its chemical instability and oral bioavailability (19.2-time increase in rats) (15). Besides, astilbin-encapsulated zein nanoparticles improved the absolute bioavailability of astilbin from 0.32 to 4.40% (16).

Nevertheless, when exposed to a certain temperature, ionic strength, and pH range, zein nanoparticles are highly susceptible to aggregation because of the strong hydrophobic attractions between them. To address the abovementioned issues, in our previous study, we used lactoferrin (LF) as a stabilizer to confirm it had good stability in fabricating zein nanoparticles at high salt concentration and wide pH range compared with other proteins. However, at low pH range, salt tolerance of zein/LF complex nanoparticles was poor (17). Thus, in our study, we want to employ the Maillard reaction to form LF-polysaccharides conjugates to overcome the above issues. The Maillard reaction is a non-enzymatic reaction that involves the condensation of the carbonyl group of a reducing carbohydrate with a free amino group of a protein (either an N-terminal amino group or a lysine residue), it is greatly accelerated by heat and alkaline conditions.

Above all, the first objective of our research was to explore the effect of selected glycosylated LF by Maillard reaction on the stability of protein-based zein nanoparticles. Subsequently, the chemical structure and microstructure of zein/glycosylated LF nanoparticles to encapsulate 7,8-DHF were explored by a series of characterization techniques. Moreover, storage stability, *in vitro* digestion, and related bioaccessibility of 7,8-DHF encapsulated nanoparticles were evaluated for potential food and pharmaceutical formulas applications.

## Materials and Methods

### Materials and Chemicals

In this study, 7,8-DHF (≥98%) was bought from TCI Co., Ltd. (Tokyo, Japan). LF (≥98%) was bought from Glycarbo Co., Ltd. (Tokyo, Japan). Pancreatin (4 × USP specification) and zein (≥95%) and was purchased from Sigma-Aldrich (MO, USA). Dextran (10, 40, and 70 kDa) and kaempferol (>98%) and was received from Aladdin Co., Ltd. (Shanghai, China). Pepsin (activity 3,000–3,500 U/mg) and bile salts and were obtained from Sangon Biotech Co., Ltd. (Shanghai, China). Other chemicals and reagents used were of analytical grade.

### Lactoferrin-Dextran Conjugates Generation *via* Maillard Reaction

Dextran (1%, w/v, 10, 40, or 70 kDa) and LF (1%, w/v) were all alone solubilized and stirred overnight at 4°C in phosphate-buffered solution (PBS, 0.01 M, pH = 7.4). Subsequently, they were blended at a 1:1 mass ratio for freeze-drying. The lyophilized mixtures proceeded Maillard reaction in a sealed desiccator containing saturated KBr solution (48 h, 60°C, and 79% relative humidity). After conjugation, the LF-dextran conjugates (LF 10K, 40K, and 70K) were lyophilized again and preserved at −20°C before study.

### Lactoferrin-Dextran Conjugates Characterization

#### Sodium Dodecyl Sulfate-Polyacrylamide Gel Electrophoresis

A 5% stacking gel and an 8% acrylamide separating gel were used for sodium dodecyl sulfate-polyacrylamide gel electrophoresis (SDS-PAGE). Furthermore, 5 μl of LF, LF 10K, LF 40K, and LF 70K solutions (according to 2 mg/ml LF) were blended with 20 μl of protein loading buffer and then heated together for 5 min in burning water. Each gel lane was loaded with 10 μl sample and performed at 80–120 mV for electrophoresis. At the end of electrophoresis, each gel was dyed with Coomassi bright blue R-250 solution (0.25%, w/v), and decolorized by decolorizing agent (50% methanol, 40% distilled water, and 10% acetic acid, v/v).

#### Conjugation Efficiency

To measure the conjugation efficiency of the samples, o-phthaldialdehyde (OPA) method was applied (18). Briefly, 4.0 ml of OPA working solution was blended with 200 μl of LF and three glycosylated LF water solutions (according to 2 mg/ml LF) after 3 min reaction at 37°C for measuring at 340 nm absorbance. The amino content standard curve was established *via* a suitable range concentration of L-leucine (0.25–2.5 mM). The conjugation efficiency was calculated by the following formula:


(1) $$\begin{array}{l} \text{Conjugation efficiency }\left(\%\right)\\ =\left(\text{1}-\frac{\text{amine groups after conjugation }\left(\text{mM}\right)}{\text{amine groups before conjugation }\left(\text{mM}\right)}\right)\times \text{ 100} \end{array}$$

#### Browning

The degree of browning caused by the Maillard reaction was assessed using a UV-vis spectrophotometer at 420 nm absorbance, LF, LF 10K, LF 40K, and LF 70K were dissolved in distilled water (according to 1 mg/ml LF) for measuring.

#### Zeta Potential

Zeta potential was characterized using a dynamic light scattering (DLS) instrument (Nano-ZS 90 analyzer, UK) at 25°C. LF, LF 10K, LF 40K, and LF 70K were dissolved in deionized water (according to 2 mg/ml LF) for determining by Smoluchowski model.

#### Circular Dichroism

Secondary structural characters of non-conjugates and conjugates under analysis (according to 0.2 mg/ml LF) were detected using a CD spectrometer (J-1500, JASCO, Tokyo, Japan). The secondary structure scanning region was 190–260 nm with 0.1 cm path length, the scanning speed was 50 nm/min, and bandwidth was 1.0 nm. The data were evaluated by Spectra Manager™ II Software equipped with CD spectrometer.

#### Fourier-Transform Infrared Spectroscopy

Pre-dried LF, LF 10K, LF 40K, and LF 70K under analysis was produced using adding 99% KBr disc and scanned on a Fourier-transform infrared (FTIR) spectrometer (Avatar 370, Nicolet, Madison, WI, USA). Spectral sweep band was ranged from 4,000 to 500 cm^−1^ at a 4 cm^−1^ resolution. The analytical results were obtained by OMNIC version 8.0 software.

### Zein/Glycosylated LF Composite Nanoparticles Preparation

To prepare zein/glycosylated LF composite nanoparticles by ASP method, zein was dissolved in 80% ethanol/water solution to prepare the stock solution (1% zein, w/v). Then, the mother liquor was quickly added into three glycosylated LF aqueous solutions (antisolvent) with a 1:3 volume ratio under continuous stirring at 800 rpm for 30 min, whereafter, ethanol was eliminated by a rotary evaporator at an appropriate temperature. As control samples, zein/LF and zein nanoparticles were prepared based on the above method using LF aqueous solution and deionized water as an antisolvent, respectively. The final concentration of zein, LF, and glycosylated LF in each nanoparticle were all 2.5 mg/ml. Final nanoparticles were reserved at 4°C for subsequent research. Zein/LF nanoparticles were named as Zein/LF, zein/glycosylated LF nanoparticles with different molecular weight dextran were denominated as zein/LF 10K, 40K, and 70K, respectively.

### Physical Stability of Zein/Glycosylated LF Composite Nanoparticles

#### pH Influence

The influence of pH on the stability of each colloidal particle was evaluated within a pH 3–9 range using either 2 M HCl or NaOH.

#### Ionic Strength Influence

Each zein colloidal particle was blended with NaCl to obtain samples within 0, 25, 50, 100, 200, and 500 mM NaCl concentrations at different pH (pH 3–9) and stored after 24 h for observing.

#### Temperature Influence

The temperature influence on the stability of each colloidal particle was tested by heating for 60 min at 95°C and then, cooled at 25°C.

#### Storage Time Influence

Each freshly zein colloidal particle was stored at room temperature for 1 month.

Particle size changes within complex dispersions were recorded using DLS at 25°C. The light intensity at a fixed scattering angle was 90 degrees, and the refractive index of water was set at 1.45.

### Encapsulation of 7,8-DHF Into Complex Particles

In the current study, 7,8-DHF encapsulation was conducted in the above methods described in section Zein/Glycosylated LF Composite Nanoparticles Preparation. Zein and 7,8-DHF were dissolved at 10:1 and 5:1 mass ratios in 80% ethanol-water solution, respectively. The mass ratio of LF or glycosylated LF to zein was 1:1 in the final reaction system. 7,8-DHF encapsulation in zein, zein/LF, and zein/glycosylated LF nanoparticles were denoted as DHF-zein, DHF-zein/LF, DHF-zein/LF 10K, DHF-zein/LF 40K, and DHF-zein/LF 70K, respectively. Loaded complex particles were reserved at 4°C, other samples were lyophilized for 48 h to conduct an in-depth study.

### Polydispersity Index, Particle Size, Zeta Potential, and Turbidity

Polydispersity index (PDI) and particle size of fresh dispersions were characterized based on section Physical Stability of Zein/Glycosylated LF Composite Nanoparticles. Zeta potential was tested according to section Zeta Potential. The turbidity of complex particles was tested at 600 nm using a UV spectrophotometer at 25°C.

### Entrapment Efficiency Determination

The entrapment efficiency (EE) of encapsulated 7,8-DHF was assessed by ultra-performance liquid chromatography (UPLC) based on our previously described method (19, 20) and then calculated depending on the following equation:


(2) $$\text{EE }\left(\%\right)=\frac{\text{loaded 7},\text{8}-\text{DHF}}{\text{initial 7},\text{8}-\text{DHF}}\times \text{100}$$

### Transmission Electron Microscopy

The 10-fold diluted freshly loaded colloidal particles were deposited on a copper grid with a 200-mesh formvar-carbon coating. Then, the samples were dried in air and dyed with 2% uranyl acetate. Transmission electron microscopy (TEM) (JEM-1200 EX, Tokyo, Japan) was performed for microscopic observation at 120 kV accelerating voltage.

### Field Emission Scanning Electron Microscope

The surface morphology of lyophilized nanoparticles was captured by using a field emission scanning electron microscope (FE-SEM) (GeminiSEM 300, ZEISS, Germany). Before analysis, a gold layer with 3–6 nm thickness was covered on the sample surfaces. The electron microscope acceleration voltage was 15.0 kV.

### Differential Scanning Calorimetry

Thermal behavior of 7,8-DHF, lyophilized glycosylated LF and freeze-dried nanoparticles were studied *via* differential scanning calorimetry (DSC) (Mettler Toledo, Zurich, Switzerland). About 2–8 mg of samples were accurately weighed and hermetically sealed in aluminum pots, an empty crucible under the same condition was used as a reference. Scanning calorimetry was performed at a range of 25–200°C in N_2_ atmosphere with a 10°C/min heating rate under 30 ml/min flow.

### X-Ray Diffraction

The crystalline characteristic of selected samples was evaluated on an X-ray diffractometer (Bruker D8, Karlsruhe, Germany). This diffractometer tube current and accelerating voltages were 40 mA and 40 kV, respectively. Soller slit and divergence slit were set at 2.5 and 0.5 degrees, respectively, the 2θ angle ranged from 5 to 90 degrees.

### Fourier-Transform Infrared

All analyzed colloidal dispersions were proceeded according to the method described in section Fourier-Transform Infrared Spectroscopy.

### Storage Stability of 7,8-DHF

Furthermore, 7,8-DHF, DHF-zein, DHF-zein/LF, DHF-zein/LF 10K, DHF-zein/LF 40K, and DHF-zein/LF 70K were conducted at 50°C for 72 h under dark and 25°C for 15 days under light. At an appropriate point-in-time, a certain amount of suspension was acquired for measuring by UPLC. The storage stability equation was figured as follow:


(3) $$\text{Retention rate }\left(\%\right)=\frac{\text{retained 7},\text{8}-\text{DHF concentration }}{\text{initial 7},\text{8}-\text{DHF concentration}}\times \text{100}$$

### *In vitro* Simulated Gastrointestinal Digestion

Based on the study of Yuan *et al. via* some amendments (21), briefly, 10 ml of simulated gastric fluid (SGF, 3.2 mg/ml pepsin and 2 mg/ml NaCl, pH = 2.5) and 10 ml of 7,8-DHF, DHF-zein, DHF-zein/LF, DHF-zein/LF 10K, DHF-zein/LF 40K, and DHF-zein/LF 70K were mingled for incubating in a 37°C water bath shaker for 60 min at 100 rpm. After SGF digestion, 10 ml of above-mentioned simulated gastric digestive fluids were modulated to pH 7.4 *via* 2 M NaOH. Ten milliliter of simulated intestinal fluid (SIF, 5 mg/ml bile salts, 4 mg/ml pancreatin, 6.8 mg/ml K_2_HPO_4_, and 8.8 mg/ml NaCl, and pH = 7.4) was blended to incubate for 120 min at same temperature and speed. Finally, the final digestive solution was centrifuged by 20,000 × g centrifugal force for 1 h, and the supernatant was collected. The bioaccessibility (%) was calculated based on the following equation:


(4) $$\begin{array}{l} \text{Bioaccessibility }\left(\%\right)\\ =\frac{\text{7},\text{8}-\text{DHF concentration in the supernatant phases}}{\text{7},\text{8}-\text{DHF concentration in the formulation}}\times \text{100} \end{array}$$

Besides, the digesta were gathered for acquiring particle size at designed point-in-time (30, 60, 120, and 180 min). And the final digesta were freeze-dried for FE-SEM observation.

### Statistical Analysis

Mean ± SD was presented *via* at least three times for all data. One-way ANOVA was utilized to assess the significant difference among groups (*p* < 0.05). Unpaired two-tailed Student's *t*-test (^***^p < 0.001, ^**^p < 0.01, and ^*^p< 0.05), was applied to analyze the significant differences between two groups. Data analysis was carried out by using the GraphPad Prism 8.0 (GraphPad Sofware, San Diego, CA, USA) and Origin 2021 (Origin Lab Co., Northampton, MA, USA).

## Results and Discussion

### Preparation and Characterization of Glycosylated LF *via* Maillard Reaction

**Figure 1:**
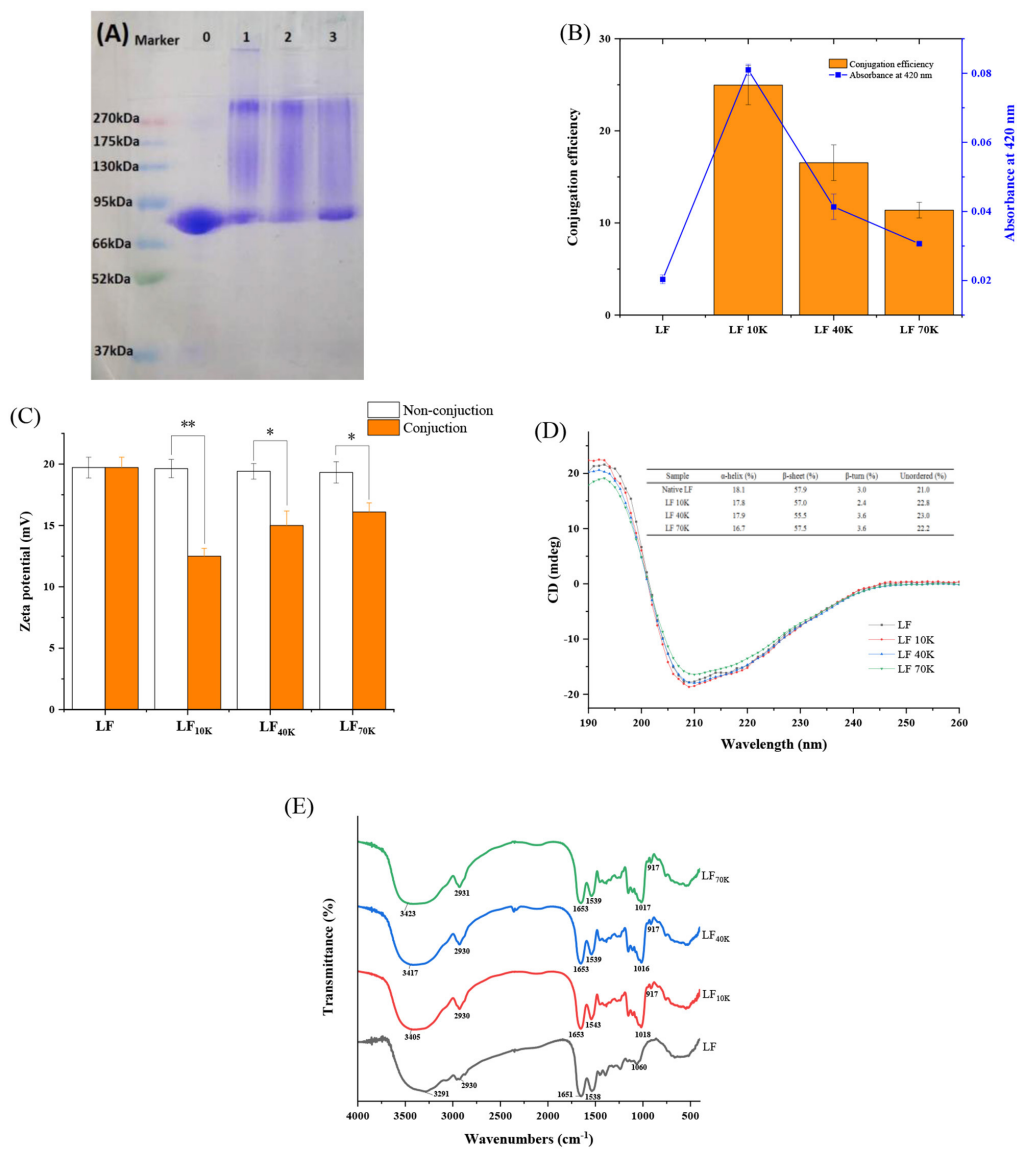
Characterization of glycosylated lactoferrin (LF) *via* Maillard reaction. sodium dodecyl sulfate-polyacrylamide gel electrophoresis (SDS-PAGE) profiles, lane 0, LF; lane 1, LF 10K; lane 2, LF 40K; lane 3, LF 70K **(A)**. Conjugation efficiency and browning **(B)**. Zeta potential, non-conjunction means the mix of LF and dextran with 10, 40, or 70 kDa **(C)**. Circular dichroism (CD) spectra **(D)**. Fourier-transform infrared (FTIR) spectra **(E)**.

As seen in Figure 1A, the molecular weight of LF and glycosylated was tested through SDS-PAGE. The major molecular weight band in native LF was at about 80 kDa (lane 0), which was in agreement with the previous literature (22). Compared with LF, after glycosylation with dextran, the bands of three LF-dextran conjugates at 80 kDa were reduced and the color became weaker, while the bands at the high molecular weight (between 85 and 270 kDa) became darker and stronger, resulting in high mobility in molecular weight. It indicated that LF and dextran generated high molecular weight glycosylated proteins (Lane 1–3) through the Maillard reaction. At the same time, it was found that the intensity and color of the bands in the high molecular weight region of LF 10K and 40K were stronger and darker than that of LF 70K, indicating that with the increase of molecular weight and the decreasing number of reductive carbonyl terminal inside dextran led to the weakening of Maillard reaction, and short polysaccharide chains were easier to conjugate to LF protein than long polysaccharide chains.

This result was confirmed by OPA results. The Maillard reaction occurs when the free amino group of the protein is covalently linked to the carbonyl group of the reducing sugar to form a Schiff base (23). As seen from Figure 1B, the grafting efficiency of LF with 10, 40, and 70 kDa dextran was about 24.96, 16.54, and 11.39%, respectively. The data certified that the molecular weight of dextran influenced the conjugation efficiency of LF. A similar result has been reported in the covalent binding of ovalbumin and dextran (18). The color depth of Maillard reaction products can intuitively reflect the degree of Maillard reaction, and the absorbance value at 420 nm is used as an indicator of the number of Browning products (24). As seen in Figure 1B, the absorbance at 420 nm of the glycosylated substances of LF to dextran with 10, 40, and 70 kDa was about 0.081, 0.041, and 0.030, respectively. Furthermore, zeta potential of glycosylated LF was significantly less than that of non-conjugated LF mixes and LF (*p* < 0.05) (Figure 1C), which was due to the positive -NH_2_ group in LF participating in the formation of Schiff base, resulting in the decrease of amount of positively charged -NH_2_ groups.

Circular dichroism spectroscopy can reflect the influence of glycosylation on the secondary structure change of LF. As observed from Figure 1D, CD scanning in the far-ultraviolet region of LF showed the negative band at 208 and 215 nm, and a maximum band at 190–195 nm, which was a typical α-helix and β-sheet structure for LF (25, 26). The inserts about secondary structure composition in Figure 1D showed that the α-helix and β-sheet of LF decreased (fall off 1.1–7.7% and 0.7–4.1% corresponding to α-helix and β-sheet, respectively), while the unordered structure increased after glycosylation (went up 5.4–8.7%), indicating that glycosylated secondary structure of LF was changed by Maillard reaction to some extent (Figure 1E). In FTIR spectroscopy, 3,300–3,600 cm^−1^ absorption peak was signified to the N-H stretching coupled with hydrogen bonding (27). The spectra of LF exhibited 3,291 cm^−1^ absorption peak, which was identical with the result of anterior research (28). As compared with LF, glycosylated LF had a significant blue-shift, and the absorption peak of LF 10K, 40K, and 70K were increased by 114, 126, and 132 cm^−1^, respectively, which implied -NH_2_ groups of LF took part in glycosylation reaction. Moreover, the major peak at 950–1,150 cm^−1^ reflected O-H deforming vibration and C-O stretching (29). We found that the absorption peak of the native LF at 1,060 cm^−1^ occurred at 42, 44, and 43 cm^−1^ redshifts corresponding to LF 10K, 40K, and 70K, respectively. As we know, dextran was rich in O-H and C-O bonds, thus illustrating reduced carbonyl group of dextran was involved in the LF Maillard reaction. In addition, after the Maillard reaction, C=O (amide I band, characteristic peak of LF was at 1,652 and 1,651 cm^−1^) and C–N stretching vibration (amide II band, characteristic peak of LF was at 1,538 and 1,538 cm^−1^) have been changed (30). Above all, the secondary and chemical structure of LF protein changes has been changed after glycosylation reaction.

### Physical-Chemical Stability Study on Zein/Glycosylated LF Nanoparticles

According to our previous research about Zein/LF (17), in our research, the selected mass ratio of glycosylated LF to zein was 1:1, and the ASP method was used to construct zein/glycosylated LF nanoparticles for the study of physicochemical stability. Zein nanoparticles and zein/LF were used as the control.

#### pH Influence

**Figure 2:**
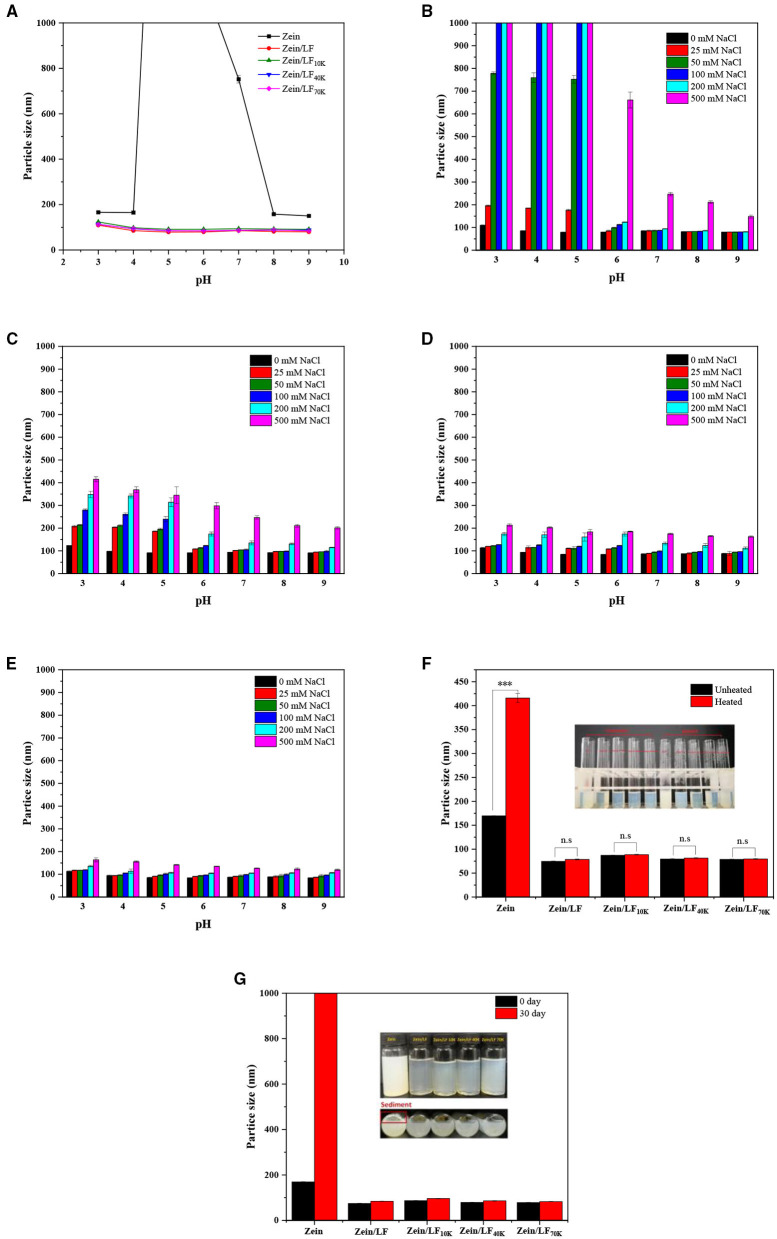
Particle size of colloidal particles at pH 3-9 condition **(A)**. Particle size of colloidal particles under 0-500 mM NaCl concentrations at pH 3-9 condition, zein/LF **(B)**, zein/LF 10K **(C)**, zein/LF 40K **(D)**, zein/LF 70K **(E)**. Particle size of colloidal particles under thermal treatments **(F)**. Particle size of colloidal particles in 1-month storage at 25°C **(G)**.

As observed from Figure 2A, our previous study has proved that bare zein nanoparticles occurred severe aggregation at pH 5.0–7.0 accompanied by a large rise in particle size. Besides, the addition of LF greatly prevented zein nanoparticles from aggregation at pH 5.0–7.0 due to that LF provided strong electrostatic repulsion and steric exclusion (17). In addition, glycosylated LF adding exhibited the same stabilizing effect compared with native LF at a pH range of 3–9, no matter what zein/LF 10K, 40K, and 70K. This might be ascribed to the shielding effect of particle surface charge by protein-carbohydrate layer. The same result was found in caseinate-carbohydrate conjugates coating zein nanoparticles (31). Above all, glycosylated LF acted as protein emulsifiers, exhibited the same advantages for zein nanoparticle stabilization at pH 3–9 compared with LF.

#### Ionic Strengths Influence at Different pH Condition

Colloidal systems will undergo different ionic environments in the commercial food products and human digestive tract. Thus, ionic strengths stability testing is necessary to evaluate the functionality of colloidal particles (Figures 2B–E) (32). Previously, it has been proved that zein unitary system is highly sensitive to ionic strength, even at a low level of salt. Although the zein/LF binary system with the addition of LF has been improved, it was still highly sensitive to the ionic strength to produce aggregation at low pH (3–5) (17). However, under the presence of LF 10K, 40K, and 70K, the particle size of zein/glycosylated LF nanoparticles maintained stable at a broad pH range of 3–9 when adding 0–500 mM NaCl, particularly zein/LF 40K and 70K. The mean particle size of zein/LF 40K and zein/LF 70K was below 250 and 200 nm, respectively, at a pH range of 3–9 when adding 0–500 mM NaCl, zein/LF 70K showed the best stabilization effect. These results showed that with the increase of the molecular weight of dextran, its chain length increased correspondingly, resulting in a greater steric hindrance, which prevented the agglomeration effect of nanoparticles. At the same time, the interface layer formed by glycosylated LF coating particle surface played a role in shielding the external charge. It is demonstrated that zein/glycosylated LF nanoparticles as nutritional ingredients will remain certainly stable in commercial products, it is also manifested that they as colloidal delivery systems may improve the stability and bioaccessibility of bioactive compounds through the human gastrointestinal tract with relatively high ionic strengths.

#### Thermal Treatment Influence

Heat treatment was often applied to sterilize and pasteurize food or beverages. As seen in Figure 2F and Supplementary Figure 2 (photograph), after heating at 95°C for 60 min, the average particle size of bare zein nanoparticles was above 400 nm. A previous study has shown that bare zein nanoparticles are highly sensitive to under heating conditions (33). However, under the same heating conditions, the particle size of zein/LF, zein/LF 10K, zein/LF 40K, and zein/LF 70K had no significant change, indicating the presence of native or glycosylated LF improved thermal stability of zein nanoparticles.

#### Long-Term Storage Influence

In daily commodity circulation, it is critical to evaluate the long-term stability of colloidal particles in room temperature circumstances. As seen from Figure 2G, individual zein nanoparticles displayed highly instability accompanied by aggregation range from pH 3.0 to 9.0 after 1-month storage. However, whatever the presence of native or glycosylated LF, the composite nanoparticles were stable to prevent aggregation for 30 days storage, demonstrating glycosylated LF coating exhibited same effect at increasing the long-term storage stability for zein nanoparticles compared with native LF.

### Encapsulation of 7,8-DHF

The influence of *via* glycosylated LF with different molecular weight (LF 10K, 40K, and 70K) and 7,8-DHF concentration on EE, PDI, particle size, zeta potential, and turbidity within different delivery systems are summarized in Table 1. Without 7,8-DHF, zeta potential of bare zein particles was 4.66 mv, the increase of positive charge occurred when supplementation with glycosylated LF, displaying 21.63–23.45 mv zeta potential, which was due to amino acid residue (-NH^3+^) in LF 10K, 40K, and 70K. But zeta potential values of zein/glycosylated LF nanoparticles were all lower than that of zein/LF, furtherly clarifying that amino acid residue in LF participated in the Maillard reaction. Homogeneous colloid systems (79.27–87.24 nm) came true by reducing the surface hydrophobicity of the zein protein when adding LF 10K, 40K, and 70K in contrast to bare zein nanoparticle (169.56 nm). In addition, compared with zein/LF, the particle size of zein/LF 10K, 40K, and 70K was larger, which was due to long polysaccharide chains conjugated to the surface of LF protein. In addition, turbidity and PDI showed a similar trend to particle size The decrease manifested that there is more glycosylated LF coating on the surface of zein nanoparticles, which inhibits their micro-aggregation. At the 7,8-DHF to zein mass ratio was 1:5, EE of zein, zein/LF, zein/LF 10K, zein/LF 40K, and zein/LF 70K was 37.27, 66.10, 72.41, 84.75, and 83.61%, respectively. Above outcomes manifested that the addition of LF glycosylation further increased EE of 7,8-DHF (*p* < 0.05), particularly LF 40K and 70K. An identical result was discovered at the mass ratio of 7,8-DHF to zein 1:10 (the EE of DHF-zein/LF 10K, 40K, and 70K were all above 98%). In general, as 7,8-DHF was embedded, it could disturb mutual effect among hydrophobic groups of zein, leading to the stability of zein particles, with the accompanying decline in particle size (116.9–119.7 nm). However, the particle size of DHF-Zein/LF 10K, 40K, and 70K slightly increased compared with unloaded nanoparticles but were all below 100 nm, displaying a small nanoscale size. In addition, 7,8-DHF encapsulation led to the increase of PDI and turbidity of nanoparticles, a homologous tendency was reported by Sun et al. (34). After the lyophilized DHF-zein/LF 10K, 40K, and 70K were redissolved in the distilled water, the redispersibility was good, the redissolved nanoparticles still maintained a good EE (above 93.43%), zeta potential (above 18.43 mV), particle size (below 108.2 nm), PDI (below 0.281), and turbidity (below 0.267) value, which was owed to that hydrophilic glycosylated LF adsorbing adequately on the surface of the zein resulted in a decrease in the surface hydrophobicity. Collectively, zein/glycosylated LF nanoparticles were a high-quality colloidal delivery for 7,8-DHF, particularly zein/LF 40K and 70K.

**Table 1:** Entrapment efficiency (EE), zeta potential, particle size, polydispersity index (PDI), and turbidity of 7,8-dihydroxyflavone (7,8-DHF) in different colloidal systems.

**(Zein or LF): 7,8-DHF (w/w)**	**Colloidal systems**	**EE (%)**	**Zeta potential (mV)**	**Particle size (nm)**	**PDI**	**Turbidity**
Without 7,8-DHF	Zein	–	4.66 ± 0.13^a^	169.6 ± 0.54^a^	0.262 ± 0.005^bc^	1.465 ± 0.05^a^
	Zein/LF	–	26.93 ± 0.33^c^	74.6 ± 0.65^c^	0.199 ± 0.008^b^	0.140 ± 0.03^c^
	Zein/LF 10K	–	21.63 ± 0.46^c^	87.24 ± 0.16^c^	0.227 ± 0.014^a^	0.219 ± 0.06^c^
	Zein/LF 40K	–	23.45 ± 0.32^c^	79.27 ± 0.96^c^	0.213 ± 0.010^b^	0.134 ± 0.03^c^
	Zein/LF 70K	–	23.17 ± 0.12^c^	78.67 ± 0.51^c^	0.205 ± 0.004^b^	0.125 ± 0.07^c^
5:1	Zein	37.27 ± 1.36^d^	10.23 ± 0.13^b^	119.7 ± 5.69^b^	0.361 ± 0.010^d^	0.451 ± 0.07^b^
	Zein/LF	66.10 ± 2.63^c^	24.14 ± 0.36^c^	84.3 ± 1.21^c^	0.384 ± 0.021^d^	0.206 ± 0.04^c^
	Zein/LF 10K	72.41 ± 2.12^c^	18.74 ± 0.16^c^	98.1 ± 1.41^c^	0.371 ± 0.014^c^	0.287 ± 0.06^c^
	Zein/LF 40K	84.75 ± 3.52^b^	20.41 ± 0.26^c^	88.41 ± 2.01^c^	0.363 ± 0.024^d^	0.174 ± 0.02^c^
	Zein/LF 70K	83.61 ± 2.88^b^	20.31 ± 0.31^c^	87.98 ± 2.16^c^	0.357 ± 0.030^d^	0.168 ± 0.03^c^
10:1	Zein	46.38 ± 2.46^d^	11.24 ± 0.14^b^	116.9 ± 1.76^b^	0.291 ± 0.020^c^	0.401 ± 0.08^b^
	Zein/LF	98.31 ± 4.12^a^	25.41 ± 0.43^c^	82.3 ± 1.01^c^	0.321 ± 0.014^d^	0.145 ± 0.05^c^
	Zein/LF 10K	98.66 ± 3.54^a^	19.74 ± 0.23^c^	95.6 ± 1.41^c^	0.345 ± 0.037^bc^	0.230 ± 0.09^c^
	Zein/LF 40K	99.41 ± 4.14^a^	21.45 ± 0.31^c^	85.79 ± 0.87^c^	0.334 ± 0.047^d^	0.138 ± 0.02^c^
	Zein/LF 70K	99.21 ± 4.14^a^	21.31 ± 0.51^c^	85.64 ± 1.26^c^	0.330 ± 0.034^d^	0.127 ± 0.06^c^
10:1	Redissolved Zein/LF	92.66 ± 3.54^a^	24.31 ± 0.32^c^	103.6 ± 1.41^c^	0.265 ± 0.037^bc^	0.165 ± 0.05^c^
	Redissolved Zein/LF 10K	93.43 ± 2.56^a^	18.43 ± 0.41^c^	108.2 ± 1.43^c^	0.273 ± 0.024^bc^	0.267 ± 0.04^c^
	Redissolved Zein/LF 40K	94.21 ± 3.21^a^	21.01 ± 0.24^c^	105.3 ± 1.75^c^	0.281 ± 0.021^bc^	0.154 ± 0.03^c^
	Redissolved Zein/LF 70K	94.25 ± 4.35^a^	21.13 ± 0.32^c^	105.7 ± 1.24^c^	0.278 ± 0.042^bc^	0.152 ± 0.03^c^

*Values are the means ± SD (n = 3). Different letters in the same column indicate significant differences (p < 0.05) based on one-way ANOVA followed by Tukey's honest significant difference post-hoc tests*.

### Micromorphology

**Figure 3:**
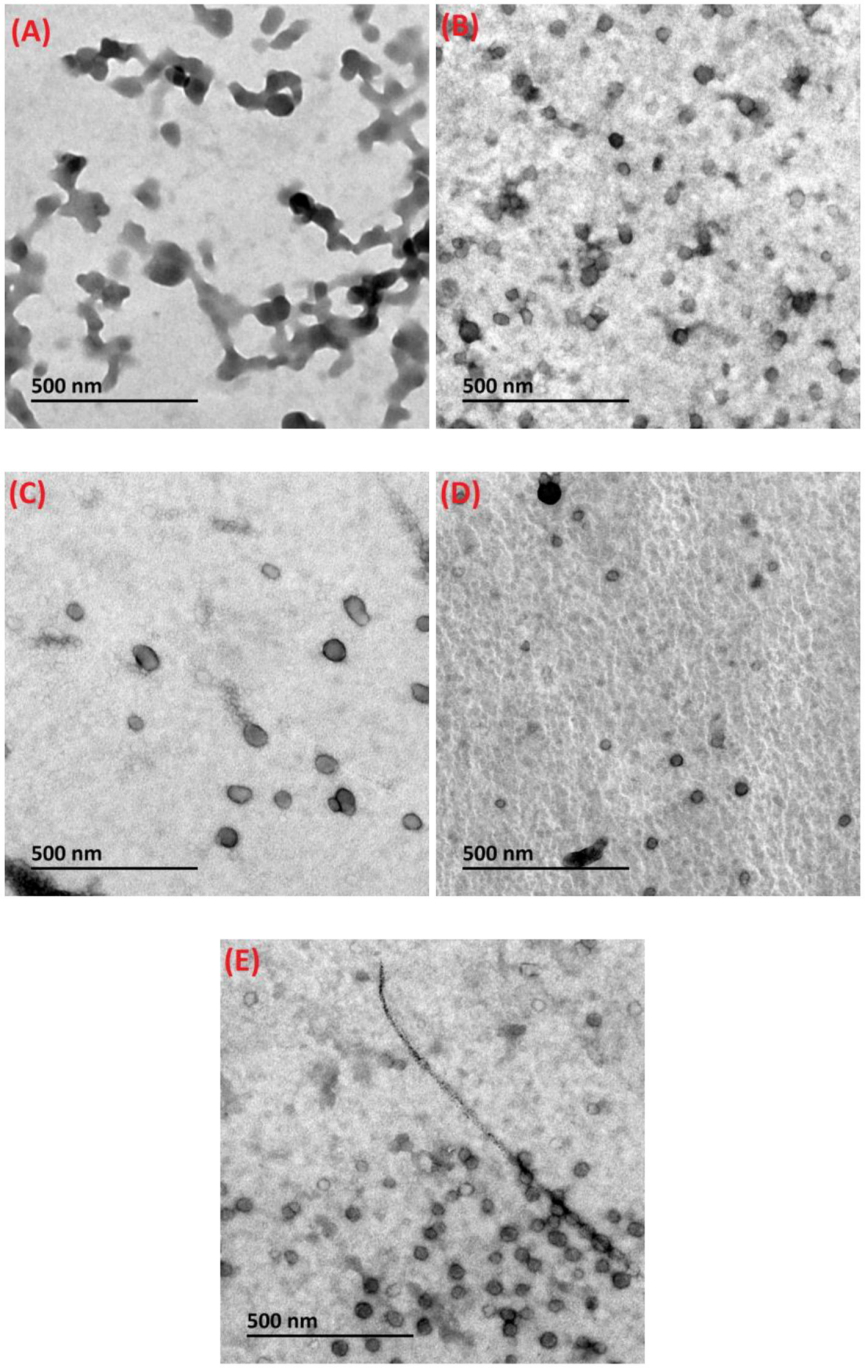
Transmission electron microscopy (TEM) images of dihydroxyflavone (DHF)-zein **(A)**, DHF-zein/LF **(B)**, DHF-zein/LF 10K **(C)**, DHF-zein/LF 40K **(D)**, and DHF-zein/LF 70K **(E)**. Pictures were taken at ×50,000 magnifications.

The microstructural features of loaded composite nanoparticles were analyzed *via* TEM. As observed from Figure 3A, the diameter of DHF-zein was above 100 nm, which was consistent with the DLS conclusions. In addition, DHF-zein was interconnected, which may be due to the hydrophobic interaction among zein particles after they were diluted 10 times, resulting in aggregation. However, after the addition of glycosylated LF, DHF-zein/LF 10K, 40K, and 70K showed a spherical shape in the range of 70–100 nm (Figures 3B–E) and were homogeneously dispersed, indicating that the adsorption of glycosylated LF could increase the electrostatic repulsion and spatial repulsion effect. Thus, the aggregation of DHF-zein could be prevented. In addition, the average particle size of DHF-zein/glycosylated LF was relatively larger than that of DHF-zein/LF, especially DHF-zein/LF 10K.

**Figure 4:**
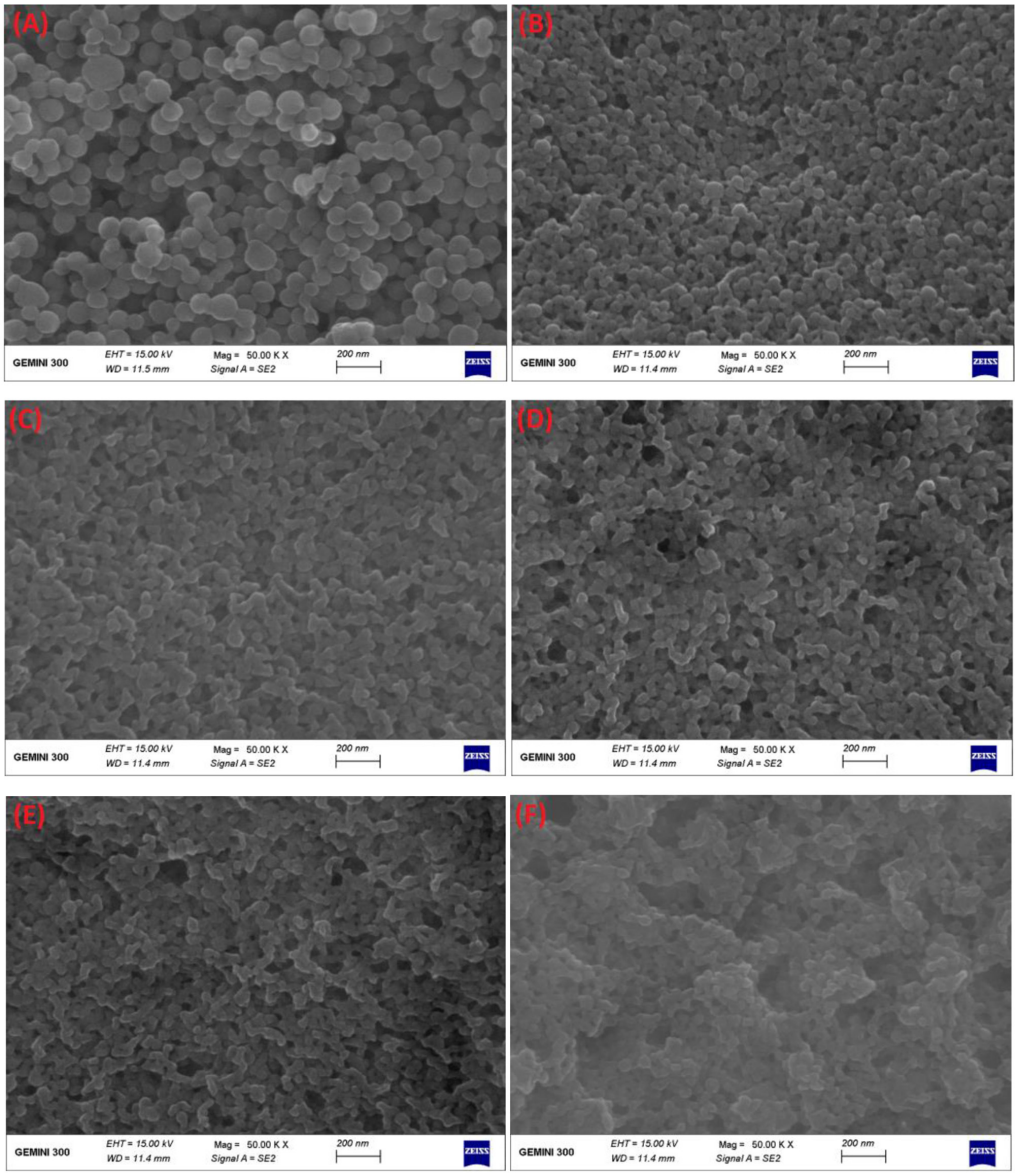
Field emission scanning electron microscope (FE-SEM) images of bare zein nanoparticles **(A)**, DHF-zein **(B)**, DHF-zein/LF **(C)**, DHF-zein/LF 10K **(D)**, DHF-zein/LF 40K **(E)**, and DHF-zein/LF 70K **(F)**. Pictures were taken at ×50,000 magnifications.

Furthermore, FE-SEM was applied to observe the differences in surface microscopic morphology of each composite nanoparticle (Figure 4). As seen from Figure 4A, zein nanoparticles and DHF-zein were typically of spherical shapes with uniform in size (35), but the surface morphology of DHF-zein emerged slightly rough, it was consistent with a preceding study about curcumin (36). However, after adding glycosylated LF, DHF-zein/LF 10K, 40K, and 70K were significantly changed with irregular and rough surfaces in comparison to zein nanoparticles and DHF-zein (Figures 4D–F). This appearance was probably due to our hypothesis that glycosylated LF was adsorbed on the surface of DHF-zein by some non-covalent forces. This result was consistent with that of native LF adsorbed on the surface of DHF-zein (Figure 4C). In addition, compared with bare zein nanoparticles, the average particle size of the binary system was decreased after encapsulating, which was consistent with the results measured by DLS.

### Differential Scanning Calorimetry

**Figure 5:**
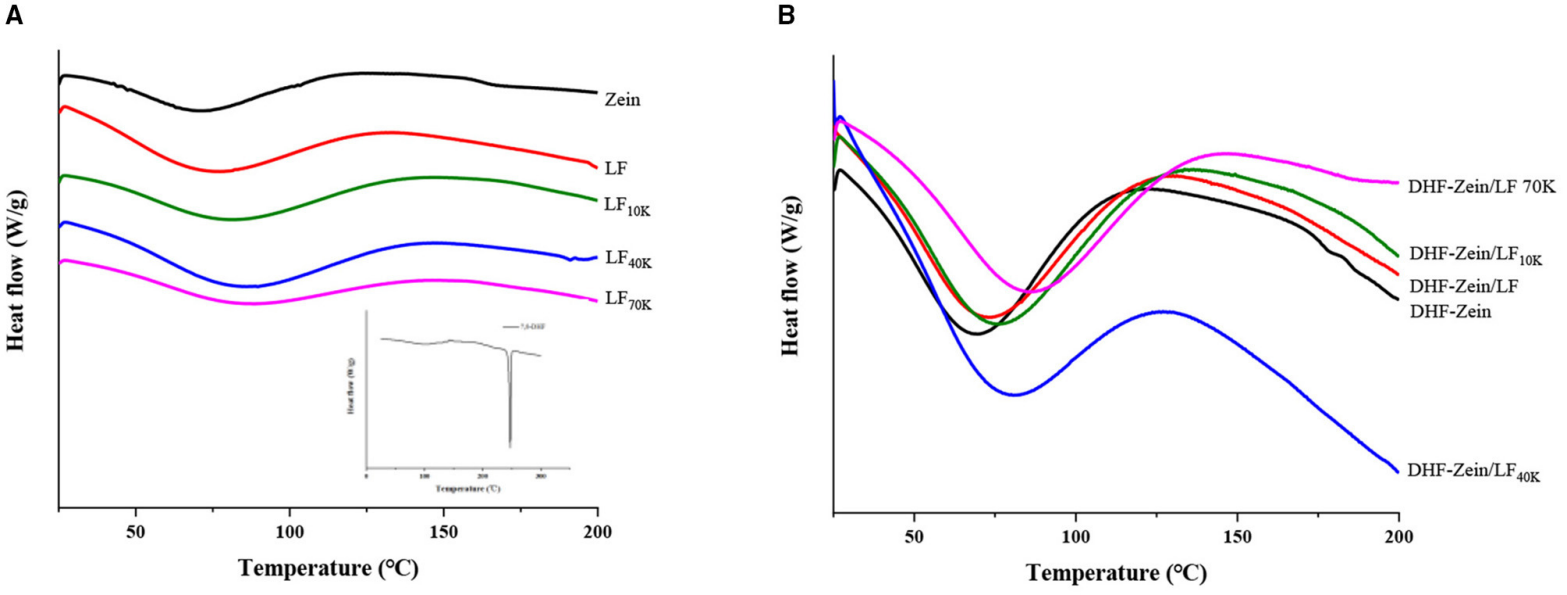
Differential scanning calorimetry (DSC) analysis of samples, such as individual zein, LF, glycosylated LF, and 7,8-DHF **(A)**, DHF-zein, DHF-zein/LF, DHF-zein/LF 10K, DHF-zein/LF 40K, and DHF-zein/LF 70K **(B)**.

As a thermal and crystallographic analysis technique, DSC was used to understand the thermal behavior associated with phase transformation of biomaterials *via* thermodynamic characterization (37). As shown in Figure 5A, for the pure 7,8-DHF, the embedded thermograms showed a narrow and sharp peak at 246.24°C, which might be attributed to the melting of 7,8-DHF crystals (38). Besides, compared with other flavonoids, such as curcumin (39), the endothermic peak of 7,8-DHF was higher. The DSC curve of zein exhibited broad endothermic peaks at around 71.12°C, this result was less than the reports of Dai et al. (40), which showed that endothermic peaks of zein were at around 81.16°C. Furthermore, the characteristic endothermal peak of LF, LF 10K, LF 40K, and LF 70K was at around 75.41, 80.56, 86.22, and 88.12°C, respectively, confirming that glycosylation reactions jointed with polysaccharides improved the thermostability of LF. After Maillard reaction, its affinity with bound water became strong (41), and the polysaccharide chain was longer, the binding water ability was stronger. After 7,8-DHF encapsulation, no distinctive endothermic peaks of 7,8-DHF in DHF-zein, DHF-zein/LF, DHF-zein/LF 10K, DHF-zein/LF 40K, and DHF-zein/LF 70K were discovered, which suggested that 7,8-DHF changed from the crystalline state to the amorphous state. Some similar observations have been reported about curcumin (42) and β-carotene (43). Besides, the endothermic peak of DHF-zein was decreased from 71.12 to 68.57°C compared with bare zein nanoparticles (Figure 5B), the decrease in melting temperatures might be attributed to intermolecular interactions between 7,8-DHF and zein. In addition, Wei et al. reported the existence of resveratrol declined the thermal stability of zein nanoparticles (44). Most intriguingly, after adding native LF and glycosylated LF, the endothermic peak of DHF-zein/LF, DHF-zein/LF 10K, DHF-zein/LF 40K, and DHF-zein/LF 70K was shifted to 72.61, 75.74, 80.89, and 85.60°C (Figure 5B), indicating that they improved the thermal stability of DHF-Zein nanoparticles, particularly LF 70K.

### X-Ray Diffraction

**Figure 6:**
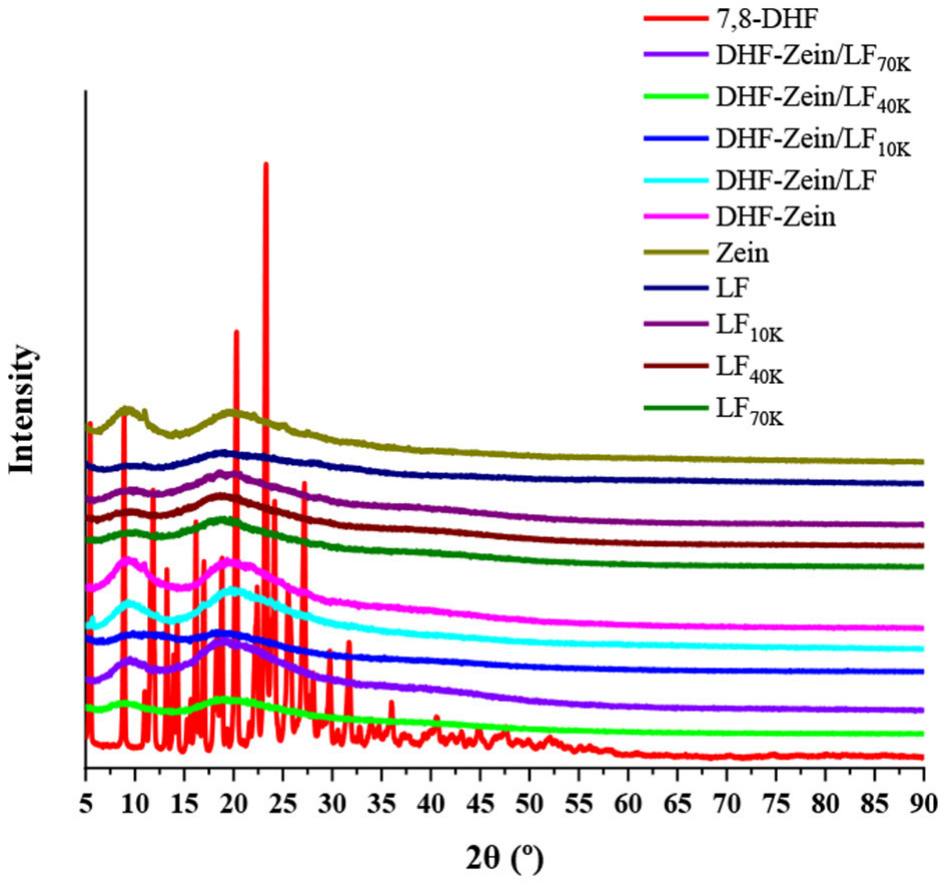
X-ray diffraction (XRD) spectra of free 7,8-DHF, zein, LF, glycosylated LF DHF-zein, DHF-zein/LF, DHF-zein/LF 10K, DHF-zein/LF 40K, and DHF-zein/LF 70K.

**Figure 7:**
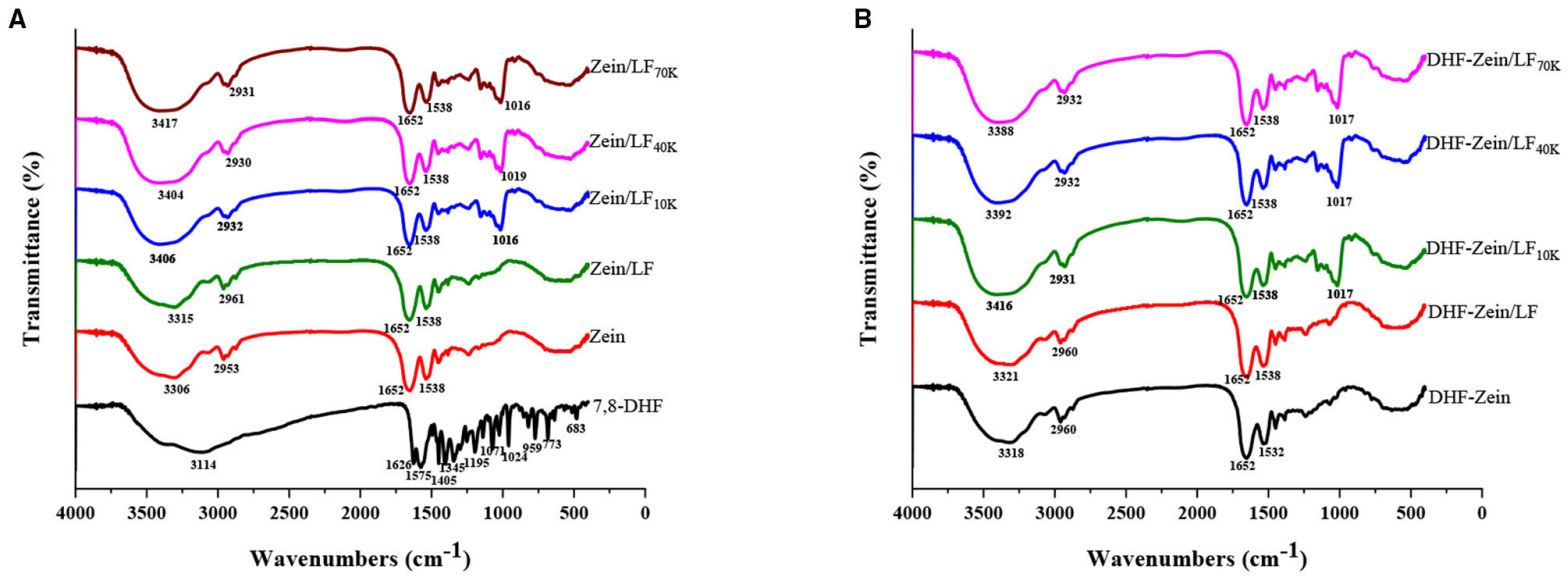
Fourier-transform infrared spectra of free 7,8-DHF, zein, zein/LF, zein/LF 10K, zein/LF 40K, zein/LF 70K **(A)**, DHF-zein, DHF-zein/LF, DHF-zein/LF 10K, DHF-zein/LF 40K, and DHF-zein/LF 70K **(B)**.

X-ray diffraction ranging from 5 to 90 degrees at 2θ values was applied to examine the physical state of 7,8-DHF in different nanoparticles. As known in Figure 6, 7,8-DHF was extremely crystalline with multiple sharp diffraction peaks at the 5–40 degrees range. In contrast, when the diffraction angles of zein and LF in the 2θ range were 9.3 and 19.6 degrees and 9.7 and 18.6 degrees, respectively, two flat humps without sharp diffraction maximum have appeared in the XRD spectrum, besides, LF 10K, 40K, and 70K had similar peak pattern to native LF, indicating that glycosylated LF were all in an amorphous form (40). However, no distinctly characteristic diffraction maximum for 7,8-DHF was found in zein/glycosylated LF composite nanoparticles. Such behavior indicated 7,8-DHF was completely loaded into zein/LF 10K, 40K, 70K, and existing in an amorphous state. This discovery confirmed the results based on DSC analysis. Furthermore, similar outcomes were discovered by previous studies about coenzyme Q10, curcumin, and resveratrol (45–47). Astonishingly, in contrast to DHF-zein, the diffraction peak of DHF-zein/LF and 70K at 19.6 degrees was remarkably increased and the peaks of DHF-zein/LF 10K and 40K at these diffraction angles almost disappeared. This result can be explained that due to changes in the interactions (hydrophobic effect, hydrogen bonding, and electrostatic interaction) along with zein, LF and glycosylated LF within the different systems, distinguishable behaviors of in DHF-zein, DHF-zein/LF, DHF-zein/LF 10K, DHF-zein/LF 40K, and DHF-zein/LF 70K were observed (44).

### Fourier-Transform Infrared

Fourier-transform infrared is a versatile tool for monitoring changes within the functional groups of biopolymers and evaluating interactive force among components post-particle formation. As shown in Figure 7, the O–H group stretching characteristic peak of zein was 3,306 cm^−1^ (Figure 7A) (48). When zein was combined with glycosylated LF to form zein/LF 10K, 40K, and 70K, the hydrogen bonds characteristic peaks were transformed from 3,306 to 3,406 cm^−1^ and 3,404 and 3,417 cm^−1^, respectively, indicating the hydrogen bonds existed in the binding of zein and glycosylated LF. Besides, the forming hydrogen bonds were stronger than that of Zein/LF. In addition, 2,953 cm^−1^ is considered to be the hydrophobic C–H group stretching vibration peak in zein (49). After the formation of zein/LF 10K, 40K, and 70K, their characteristic peaks redshifted from 2,953 to 2,932 cm^−1^ and 2,930 and 2,931 cm^−1^, indicating that stronger hydrophobic interactions existed in the formation of zein/glycosylated LF nanoparticles compared with Zein/LF. According to Liu et al. report (35), the 1,652 cm^−1^ peak of zein was the C=O stretching (amide I). In addition, 1,538 cm^−1^ peak was primarily associated with the bending of N–H coupled with the stretching of C–N (amide II). With LF 10K, 40K, and 70K incorporation, the amide I and amide II characteristic peaks of zein/glycosylated LF nanoparticles had no change, elucidating that zein/glycosylated LF nanoparticles formation was not involved in electrostatic interaction (46). Additionally, hydrophobic interaction and hydrogen bonds among glycosylated LF and zein were confirmed by the result of DSC about the increasing endothermic peak temperature of nanoparticles.

As shown in Figure 7A, the peaks at 3,114, 1,626, 1,575, 1,405, 1,195, and 1,071 cm^−1^ were the typical peaks of 7,8-DHF, which has been confirmed in our foregoing research (11). Expectedly, these characteristic peaks of 7,8-DHF have vanished in DHF-zein, DHF-zein/LF, DHF-zein/LF 10K, DHF-zein/LF 40K, and DHF-zein/LF 70K, indicating that these nanoparticles successfully encapsulated for 7,8-DHF. Moreover, compared with the unloaded nanoparticles, the wavenumbers of the main characteristic peaks of the loaded nanoparticles were all changed, indicating that the presence of 7,8-DHF could also change the non-covalent binding ability of the composite carriers to some extent.

### A Graphic Illustration for the Formation and Stability Mechanism of Nanoparticles

**Figure 8:**
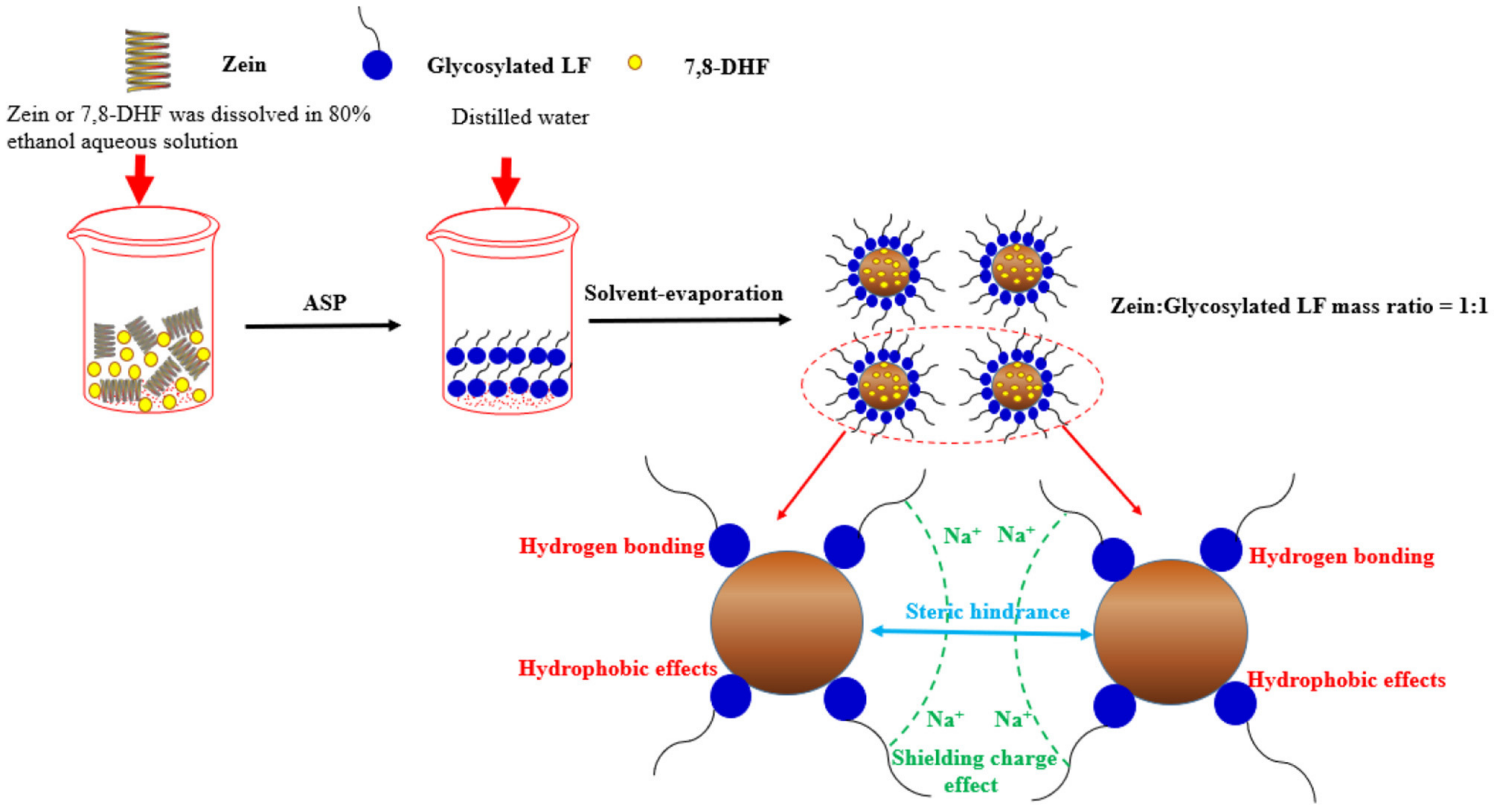
An illustration of the formation and stability mechanism of DHF-zein/glycosylated LF.

Diverse technologies, such as EE, PDI, particle size, zeta potential, turbidity, TEM, FE-SEM, DSC, XRD, and FTIR were applied to make clear formation and stability mechanism of zein/glycosylated LF delivery system (Figure 8). After zein was rapidly added into glycosylated LF (mass ratio 1:1), sufficient glycosylated LF acted as a shielding effect (electrostatic repulsion) and steric hindrance stabilizer was coated onto the surface of zein particles to stop their sedimentation, showing a low PDI, turbidity, and size based on DLS and UV. Especially in the high ionic strengths (0–500 mM NaCl) at wide pH 3–9 range condition, glycosylated LF played a stabilizing role *via* above-mentioned shielding effect and steric hindrance. And some internal drives (hydrogen bonding and hydrophobic effect) participated in the formation of zein/glycosylated LF nanoparticles according to FTIR and FE-SEM. Employing EE, XRD, DSC, TEM analysis, 7,8-DHF was successfully encapsulated in zein/glycosylated LF nanoparticles with relatively uniform sphericity, displaying a good entrapment efficiency.

### Storage Stability of 7,8-DHF

**Figure 9:**
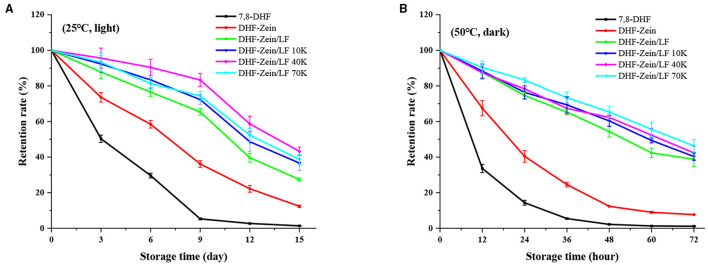
Storage stability of free 7,8-DHF, DHF-zein, DHF-zein/LF, DHF-zein/LF 10K, DHF-zein/LF 40K, and DHF-zein/LF 70K at 25°C under light **(A)** and at 50°C under dark **(B)**.

To prevent food nutraceuticals from degradation by heat or light exposure is challenging but critically necessary during storage. To meet this application end, short and long storage were investigated under varying storage environments for DHF-zein/LF 10K, 40K, and 70K. As exhibited in Figure 9A, free 7,8-DHF was mostly degraded at 25°C with light exposure for 15 days post-storage. Encapsulation of the 7,8-DHF in zein nanoparticles strengthened the storage stability (12.35%) of 7,8-DHF. The addition of LF and glycosylated LF further significantly enhanced the stability of encapsulated 7,8-DHF, in particular glycosylated LF, among them, the retention percentage of DHF-Zein/LF 40K was highest (43.21%). As seen from Figure 9B, at 50°C under dark circumstances, a similar effect was observed. The active groups of 7,8-DHF were possibly protected within the hydrophobic lumen of zein/glycosylated LF nanoparticles as a mechanism (50), especially DHF-zein/LF 70K (46.33%). The above results were in conformance to preceding studies that introduced curcumin being embedded in zein and quaternized chitosan complexes, along with work showing that quercetagetin was loaded within the zein-hyaluronic acid binary complex. Generally, the smaller the particle, the more susceptible it is to environmental factors, such as oxygen, light, and temperature. Therefore, the higher chemical stability of 7,8-DHF encapsulated in zein/glycosylated LF colloidal systems was probably due to the antioxidant activity of Maillard reaction products, which inhibited the chemical degradation of 7,8-DHF during storage. Above all, the existence of glycosylated LF enhanced the storage stability of 7,8-DHF.

### *In vitro* Simulated Gastrointestinal Digestion

**Figure 10:**
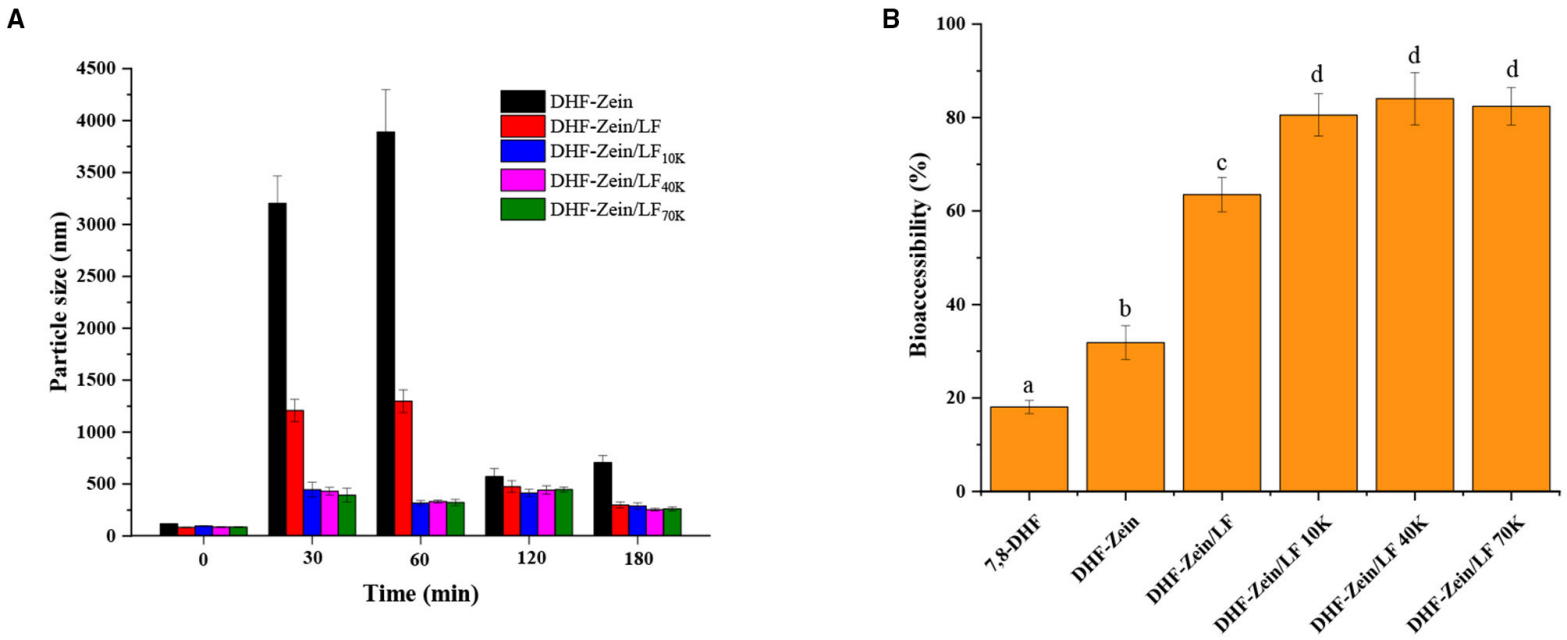
Influence of *in vitro* digestion time on the particle size of DHF-zein, DHF-zein/LF, DHF-zein/LF 10K, DHF-zein/LF 40K, and DHF-zein/LF 70K **(A)**. *In vitro* bioaccessibility **(B)**.

Effective protection of encapsulated nutritional ingredients throughout the gastrointestinal tract (GIT) is critical to assess carrier systems. In this study, an *in vitro* GIT model was applied to explore the digestive fate and bioaccessibility of 7,8-DHF in nanoparticles. Particle size changes for colloidal carriers were monitored at a specific digestion time (30, 60, 120, and 180 min), and the results were observed in Figure 10A, the mean particle size of DHF-zein was increased markedly after 60 min SGF digestion compared with original particle size (*p* < 0.05). This finding was possibly due to that the nanoparticles were subjected to ionic strength along with low pH and partially digested *via* pepsase. The low pH and ionic strength exposure likely weakened electrostatic repulsion forces among zein nanoparticles. Besides, its particle size reduction in DHF-zein post-SIF-exposure was ascribed to the fact that SIF contained bile salt, a compound with strong emulsifying abilities. Bile salt can combine many biopolymer molecules and contribute to bridging flocculation (48). An identical consequence was observed by Zou et al. report for curcumin loaded in zein nanoparticles (51). Similarly, as depicted in Figure 10A, DHF-zein/LF occurred aggregation in the SIF digestion, this result was in accord with that of pH and salt stability, which was due to the attenuated electrostatic repulsion among them. But its particles were smaller than DHF-zein after SIF digestion. Most importantly, under the presence of glycosylated LF, the mean particle size of DHF-zein/LF 10K, 40K, and 70K was mildly increased after SGF digestion, then remained fixedness during SIF digestion compared with DHF-zein and DHF-zein/LF, illustrating that the presence of glycosylated LF improved the intestinal instability of these nanoparticles. This might be due to that the glycosylated LF coating on the nanoparticles provided a greater steric repulsion that conquers attractive force (such as hydrophobic interactions or Van der Waals), which aroused the interfacial layer surrounding the colloids by shielding effect against the ionic strength and enzyme. Supplementary Figure 3 was about particle size distribution of each sample in origin, SGF and SIF digestion. In the SGF digestion, DHF-zein and DHF-zein/LF were mainly distributed in the range of 4,000–10,000 nm and 1,000–2,000 nm, respectively, and DHF-zein/glycosylated LF was mainly distributed in the range of 100–1,000 nm. Nevertheless, in the SIF digestion, particle size distribution of each sample tended to accordance, but there was bimodal particle size distribution with DHF-zein and DHF-zein/LF above 5,000 nm.

**Figure 11:**
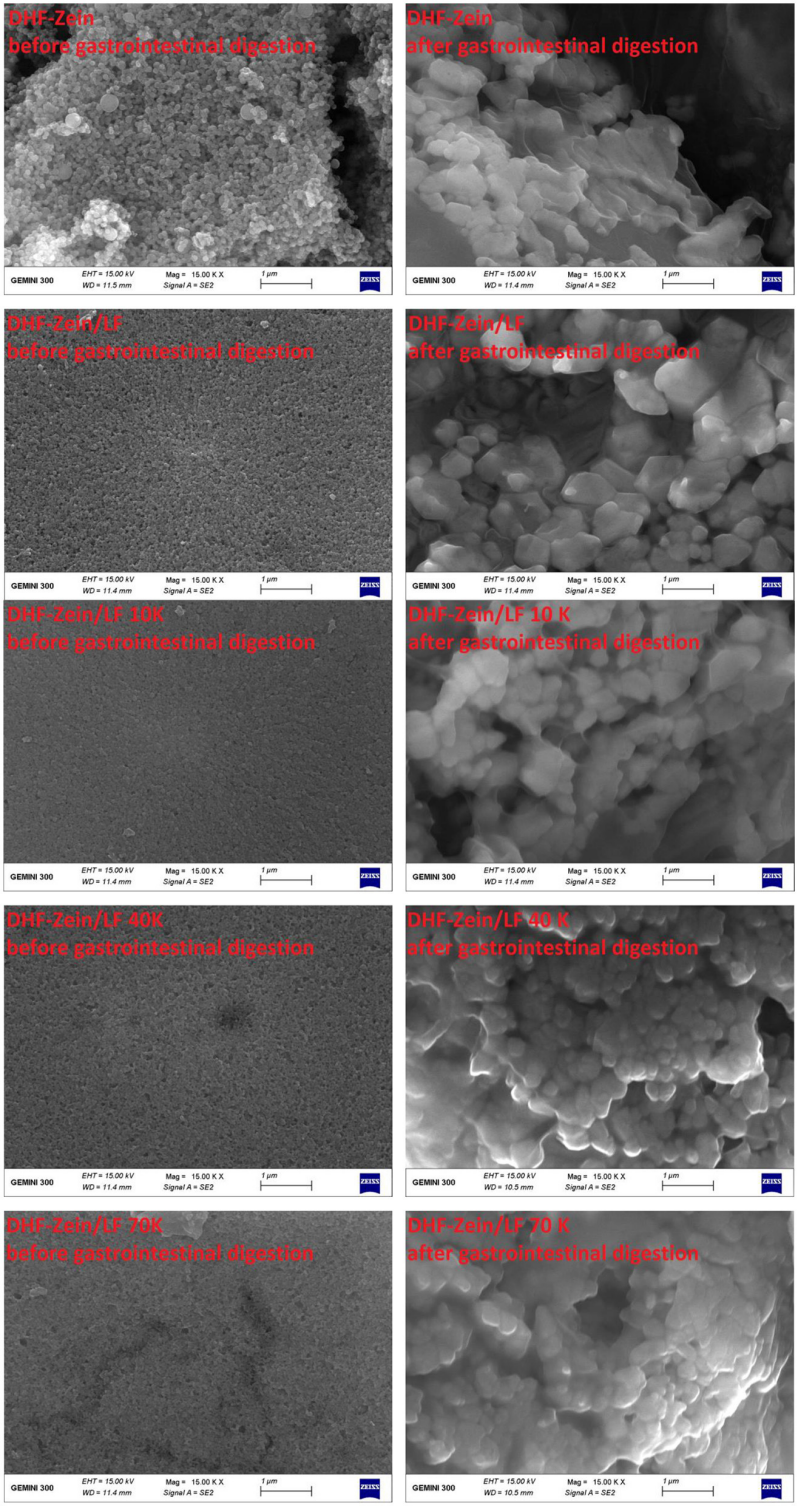
Influence of *in vitro* digestion on the FE-SEM images of DHF-zein, DHF-zein/LF, DHF-zein/LF 10K, DHF-zein/LF 40K, and DHF-zein/LF 70K. Pictures were taken at ×15,000 magnifications.

The FE-SEM microscopic observation furtherly confirmed that subjecting to GIT has a significant effect on the morphology of 7,8-DHF-loaded nano-complexes (Figure 11). DHF-zein had a spherical shape in initial conditions, after *in vitro* GIT, no spherical particle and a plate-like structure were found. This finding was unanimous to the conclusions of the anterior study (52). For DHF-zein/LF, after gastrointestinal digestion, their form changed from spherical to rectangular, this was because of a lot of aggregation in SGF digestion. However, it was amusing to notice a notably different morphology for DHF-Zein/glycosylated LF after digestion especially DHF-zein/LF 40K and 70K. They exhibited a relatively spherical morphology just like the cross-linked structure of large nanoparticles. Above all, glycosylated LF could effectively protect 7,8-DHF in delivery systems throughout GIT.

After being exposed to simulated gastrointestinal conditions, the bioaccessibility of 7,8-DHF was assayed after centrifugation and collection of micelle phases. As exhibited in Figure 10B, as its crystalline characteristic, the solubility of free 7,8-DHF was relatively low, its bioaccessibility was about 18.06% after digestion. As expected, the bioaccessibility of DHF-zein was raised to 31.85% (*p* < 0.05). In the presence of LF, its bioaccessibility increased to about 63.51%, there was a three-fold increase in comparison with free 7,8-DHF (*p* < 0.05). Most importantly, in the existence of glycosylated LF, the bioaccessibility of DHF-zein/LF 10K, 40K, and 70K all reached above 80%, particularly DHF-zein/LF 40K reached the maximum (84.05%). The higher bioaccessibility of encapsulated 7,8-DHF may be due to the following points. First, the amorphous form of 7,8-DHF is known to have a higher bioaccessibility and solubility than its crystalline form (53). Second, the partial digestion of glycosylated LF may lead to water-soluble peptides generation that can solubilize and bind to 7,8-DHF. Third, the surface of long-chain polysaccharides through steric hindrance and external charge shielding protected particles from ionic strength, enzymolysis, and interattraction. Above all, our results displayed that encapsulating 7,8-DHF in zein/glycosylated LF nanoparticles can promote a considerable increase in its *in vitro* bioaccessibility.

## Conclusions

In the present study, three zein/glycosylated LF composite spherical nanoparticles (78.67–87.24 nm) were successfully fabricated by ASP method with low PDI (<0.230) and turbidity (<0.220) values. Glycosylated LF could result fully stabilize zein nanoparticles against precipitation or aggregation, exhibiting high stability to salt at a wide pH range of 3.0–9.0. Besides, zein/glycosylated LF exhibited good thermostability and long-term storage, particularly zein/LF 40K and 70K. These zein/glycosylated LF nanoparticles were formed by hydrophobic interactions and hydrogen bonding and successfully used to encapsulate hydrophobic 7,8-DHF through DSC, EE, FIRT, and XRD. Furthermore, zein/glycosylated LF nanoparticles had good redispersibility and increase EE of 7,8-DHF, the encapsulation of 7,8-DHF improved its short- or long-term storage stability. Most astonishingly, 7,8-DHF loaded in zein/glycosylated LF nanoparticles promoted its *in vitro* bioaccessibility, particularly DHF-zein/LF 40K. Overall, these results indicate that zein/glycosylated LF nanoparticles are efficient at encapsulating, retaining, and delivering 7,8-DHF and may therefore be utilized in dietary supplements and functional foods.

## Data Availability Statement

The original contributions presented in the study are included in the article/Supplementary Material, further inquiries can be directed to the corresponding author/s.

## Author Contributions

YC: conceptualization, methodology, resources, funding acquisition, and writing—original draft preparation. XG: software and formal analysis. LW and YS: investigation and data curation. QC: project administration. SL, YW, and GX: validation, writing—review and editing and supervision. All authors have read and agreed to the published version of the manuscript.

## Funding

The authors are grateful for the grant provided by the Zhejiang Province Public Welfare Technology Application Research Project—National Cooperation Project (Grant Number LGJ21C20001), the Fund of Key Laboratory of Aquatic Product Processing, Ministry of Agriculture and Rural Affairs, China (Grant Number NYJG202104), the Fund of Guangdong Provincial Key Laboratory of Aquatic Product Processing and Safety (Grant Number GDPKLAPPS2101), the Guangxi Natural Science Foundation Program (grant number 2018GXNSFBA294015), the Zhejiang Province Xinmiao Talents Program (Grant Number 2021R403035), and the National College Students Innovation and Entrepreneurship Training Program (Grant Number 2021052 and 2021011).

## Conflict of Interest

The authors declare that the research was conducted in the absence of any commercial or financial relationships that could be construed as a potential conflict of interest.

## Publisher's Note

All claims expressed in this article are solely those of the authors and do not necessarily represent those of their affiliated organizations, or those of the publisher, the editors and the reviewers. Any product that may be evaluated in this article, or claim that may be made by its manufacturer, is not guaranteed or endorsed by the publisher.
