# Supplementary material for: Establishment and Characterization of Stable Zein/Glycosylated Lactoferrin Nanoparticles to Enhance the Storage Stability and in vitro Bioaccessibility of 7,8-Dihydroxyflavone
Source: Front Nutr. 2022 Jan 3;8:806623. doi: 10.3389/fnut.2021.806623 (PMC8763018; doi:10.3389/fnut.2021.806623)
Supplement: Supplementary file 1 [file Data_Sheet_1.docx]

**Establishment and characterization of stable zein/glycosylated lactoferrin nanoparticles to enhance the storage stability and *in vitro* bioaccessibility of 7,8-dihydroxyflavone**


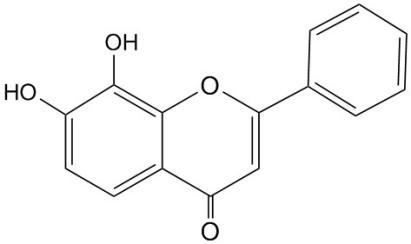


**Supplementary Fig.1** The chemical structure of 7,8-DHF


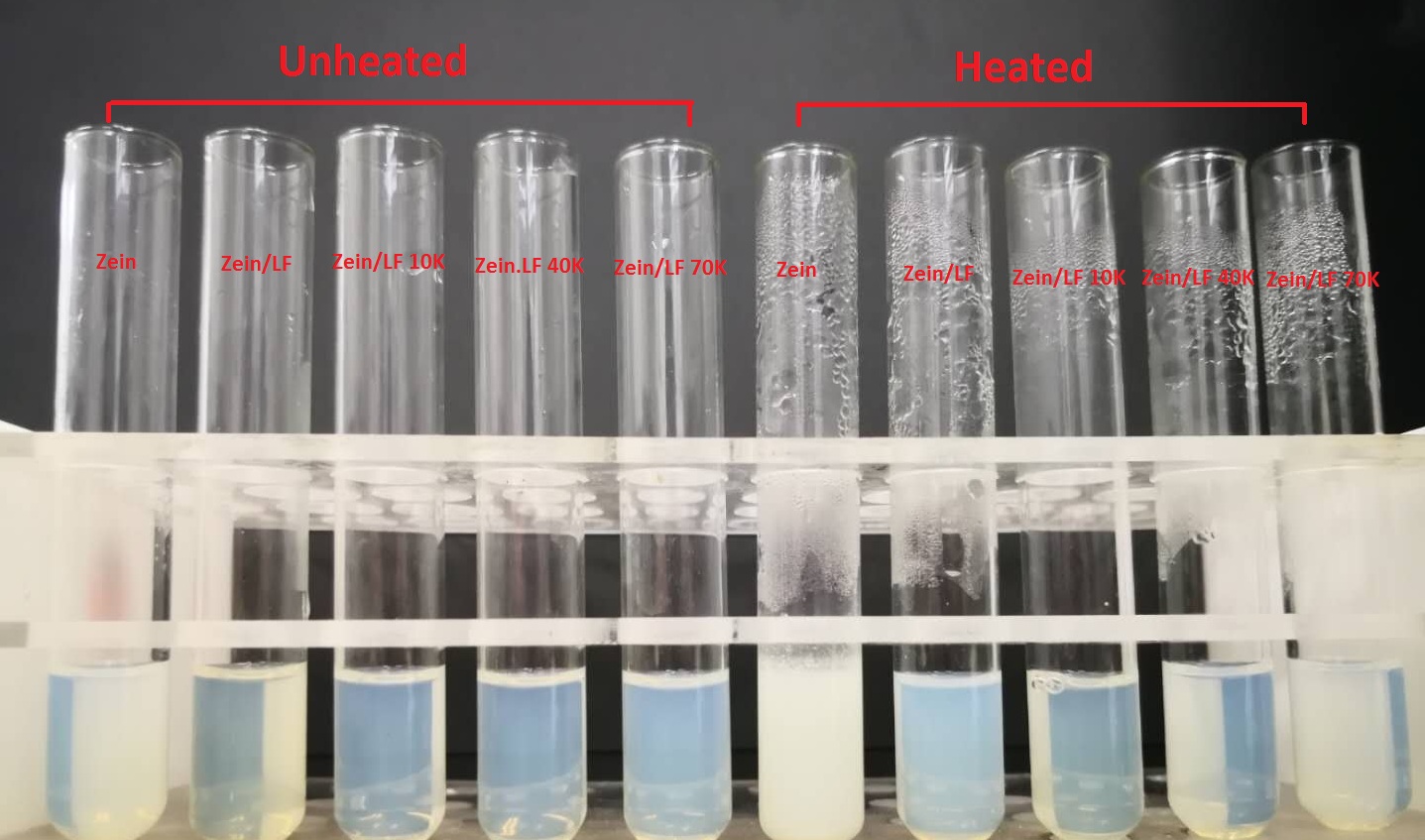


**Supplementary Fig.2** Appearence of colloidal particles under thermal treatments

**Supplementary Fig.3** Size distributions of DHF-Zein, DHF-Zein/LF, DHF-Zein/LF 10K, DHF-Zein/LF 40K and DHF-Zein/LF 70K for origin (A), SGF digestion (B) and SIF digestion (C).
